# Tetracycline Regulator Expression Alters the Transcriptional Program of Mammalian Cells

**DOI:** 10.1371/journal.pone.0013013

**Published:** 2010-09-24

**Authors:** Hubert Hackl, Anna Rommer, Torsten A. Konrad, Christine Nassimbeni, Rotraud Wieser

**Affiliations:** 1 Biocenter, Section for Bioinformatics, Innsbruck Medical University, Innsbruck, Austria; 2 Clinic of Medicine I, Medical University of Vienna, Vienna, Austria; 3 Institute of Human Genetics, Medical University of Vienna, Vienna, Austria; University of Illinois at Chicago, United States of America

## Abstract

**Background:**

Tetracycline regulated ectopic gene expression is a widely used tool to study gene function. However, the tetracycline regulator (tetR) itself has been reported to cause certain phenotypic changes in mammalian cells. We, therefore, asked whether human myeloid U937 cells expressing the tetR in an autoregulated manner would exhibit alterations in gene expression upon removal of tetracycline.

**Methodology/Principal Findings:**

Microarray analyses revealed that 172 and 774 unique genes were significantly differentially expressed by at least 2- or 1.5-fold, respectively, when tetR expressing U937 cells were maintained in media with or without the antibiotic.

**Conclusions/Significance:**

These alterations in gene expression are likely to contribute to the phenotypic consequences of tetR expression. In addition, they need to be taken into consideration when using the tetR system for the identification of target genes of transcription factors or other genes of interest.

## Introduction

Inducible ectopic expression is a widely used tool to study gene function. Its advantages compared to constitutive approaches are that early consequences of the presence of the investigated gene product can be studied, and that it facilitates the analysis also of growth inhibitory or cytotoxic gene products. Initial methods for inducible gene expression were based on mammalian promoters that could be activated, e.g., by metal ions. In the early 1990s, the adaptation of the bacterial tetracycline repressor/operator system for use in mammlian cells provided, for the first time, the possibility to regulate the expression of exogenous genes through a small molecule ligand that per se was expected not to affect the physiology of mammalian cells due to their lack of endogenous receptors for it [1]. In fact, mammalian cells need to be made responsive to tetracycline by introduction of one of several modified versions of the bacterial tetracycline regulator (tetR). The transcriptional activity of some of these derivatives is activated, and that of others repressed, by tetracycline or related compounds [1], [2]. In either case, the expression of genes cloned downstream of a promoter containing several copies of the bacterial tetracycline operator (tetO) can be regulated by removal or addition of tetracycline or tetracycline derivatives from/to the culture media. In further improvements of the system, tetR was placed under the control of tetO elements to create an autoregulatory loop, thus avoiding problems caused by toxicity of constitutive tetR expression and reducing background levels of the gene of interest in the repressed state [3], [4].

Tetracycline regulated gene expression has also been used to study the function of oncogenes involved in myeloid leukemia. A popular model for this purpose is the human cell line U937, which is able to differentiate into the monocytic lineage if stimulated with appropriate agents [5]–[16]. In the course of such studies, we and others have observed that control cells expressing only a tetR derivative, or containing a tetR derivative plus the tetO vector without a gene of interest, exhibited certain biological responses, e.g. reduced proliferation or differentiation in the absence of exogenous stimuli, to changes in the concentration of tetracycline [5], [6], [10], [12], [15].

Many leukemogenic oncogenes are transcription factors that are either aberrantly expressed or functionally altered as a consequence of chromosome rearrangements. U937 derivatives expressing such oncogenes in a tetracycline regulable manner have also been used in several microarray studies aimed at the identification of their downstream target genes [8], [14], [16]. Since, however, tetracycline removal also modifies the cellular behaviour of control U937 cells containing only the tetR but no gene of interest, we set out to ask whether these phenotypic alterations would be related to transcriptional changes. Indeed, microarray analyses revealed that several hundred genes were deregulated in tetR expressing U937 cells upon removal of tetracycline.

## Results

### Microarray analysis of U937 cells containing an autoregulated tetR

The cell line U937T_pUHD10S contains a plasmid coding for an autoregulated tetracycline repressor as well as the empty pUHD10S vector, which drives tet-regulated expression of its cDNA inserts. It was generated as a control for cell lines expressing a gene of interest from pUHD10S. Three replicate cultures of U937T_pUHD10S cells were transferred to media with or without tetracycline. 48 h later RNA was extracted and used for hybridization of Affymetrix U133 Plus 2.0 arrays. For statistical evaluation, different levels of stringency were applied so as to reflect stringency levels commonly found in the literature [8], [14], [16]. 1191 probe sets (774 unique genes) were deregulated at least 1.5-fold at p<0.05 upon removal of tetracycline from U937T_pUHD10S cells (Tables 1 and S1A). If the threshold for differential expression was increased to twofold and only genes with a false discovery rate (FDR) <10% after multiple hypothesis testing were considered, 90 probe sets (99 unique genes) resulted as deregulated (Tables 1 and S1B).

**Table 1:** Numbers of unique genes and probe sets deregulated in U937T_pUHD10S cells upon removal of tetracycline.

		FC≥1.5 p<0.05	FC≥2 p<0.05	FC≥1.5 FDR<10%	FC≥2 FDR<10%
unique genes	upregulated	465	150	115	87
	downregulated	309	22	46	12
	total	774	172	161	99
probe sets	upregulated	729	188	122	82
	downregulated	462	31	40	8
	total	1191	219	162	90

FC, fold change; FDR, false discovery rate according to Benjamini and Hochberg [20]. Because fewer hypotheses were tested when only unique genes rather than all probe sets were considered, more unique genes than probe sets may pass the multiple hypothesis test in some cases.

### Confirmation of the regulation of selected genes by real time quantitative (RTQ-) RT-PCR

**Figure 1:**
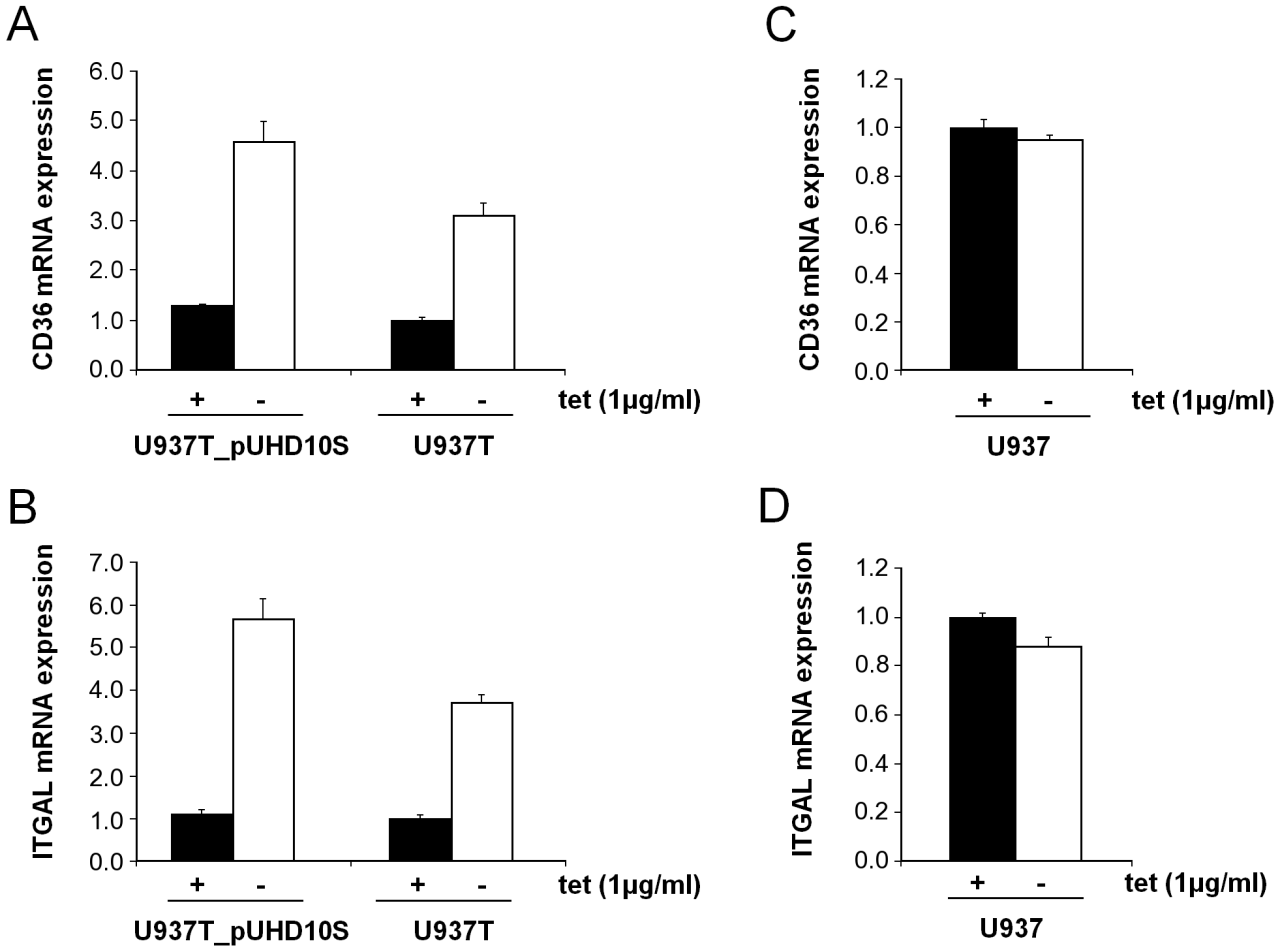
RTQ-RT-PCR confirms the induction of *CD36* and *ITGAL* upon tetracycline withdrawal from U937 cell derivatives expressing the tetR. A,B) U937T_pUHD10S cells and their parental cell line U937T, which contains the tetR but not the pUHD10S vector, were transferred to media with (black columns) or without (white columns) tetracycline (tet). After 48 h, RNA was extracted and subjected to RTQ-RT-PCR. The expression of *CD36* (A) and *ITGAL* (B) relative to the housekeeping gene *cyclophilinD* was calculated using the ΔΔC_T_ method [19]. C,D) *CD36* (C) and *ITGAL* (D) expression in native U937 cells transferred to media with or without tetracycline after being cultured in the presence of the antibiotic for one week.

Of the genes deregulated in response to tetracycline withdrawal, *CD36* (NM_000072; coding for the thrombospondin receptor) and *ITGAL* (NM_002209; coding for alpha L integrin) were selected for confirmation by RTQ-RT-PCR. Both genes form part of several gene ontology categories that were significantly enriched among genes induced by tetracycline removal (see below). RTQ-RT-PCR indeed corroborated the upregulation of these genes 48 h after removal of tetracycline from the culture media of U937T_pUHD10S cells. Both genes were also induced upon withdrawal of tetracycline from U937T cells, which contain the tetR, but not pUHD10S (Fig. 1A,B). A similar, albeit smaller and less reproducible, effect was already present 24 h after transfer of the cells to tetracycline free media (data not shown). To ask whether the changes in the expression of these genes were related to the induction of the autoregulated tetR, or represented an endogenous response of mammalian cells to altered tetracycline concentrations, native (i.e., untransfected) U937 cells were cultured in the presence of tetracycline for one week. Then they were washed and transferred to media with or without tetracycline in the same manner as U937T_pUHD10S cells. Neither *CD36* nor *ITGAL* was induced upon transfer of native U937 cells to tetracycline free media (Fig. 1C,D).

### Gene ontology analysis

**Figure 2:**
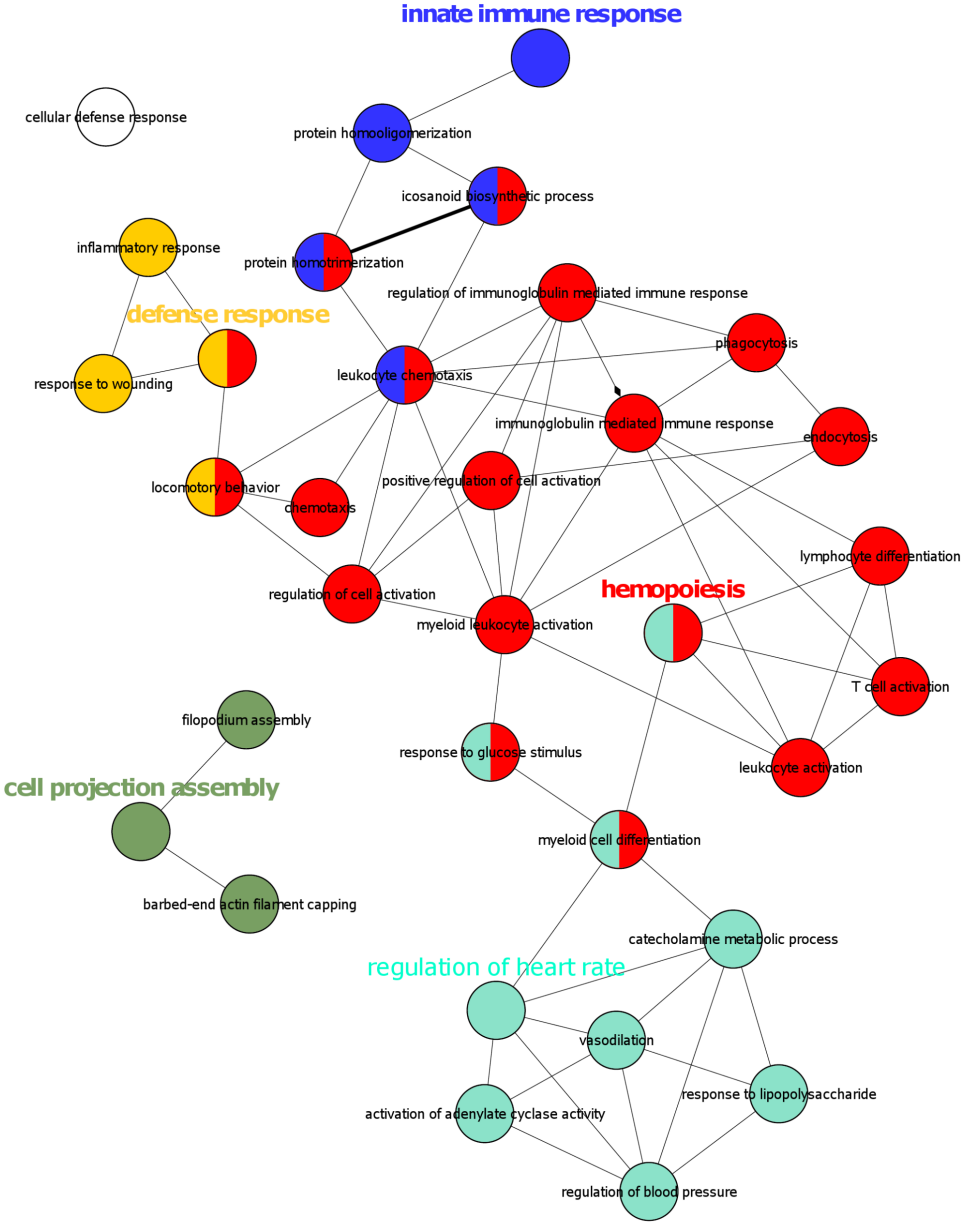
ClueGO analysis [**22**] of genes upregulated ≥1.5-fold at an FDR <10% in U937T_pUHD10S cells upon tetracycline withdrawal. Nodes (circles) represent gene ontology terms. Connections between two nodes (edges) indicate that two gene ontology terms share genes from the considered dataset (agreement measure kappa >0.3). The calculated kappa score is also used for defining functional groups, which are indicated by the same color. The most prominent gene ontology term for each group is highlighted.

Gene ontology (GO) analysis revealed the terms "hemopoiesis", "myeloid cell differentiation", "leukocyte activation", "phagocytosis", "response to stress", "defense response", "immune system process", "inflammatory response", "innate immune response", "cell activation", "signal transduction", "chemotaxis", as well as several others, to be significantly enriched among the genes upregulated at least 1.5-fold at an FDR <10% in response to tetracycline withdrawal (Fig. 2, Table S2).

## Discussion

Because we and others have found that tetR expressing cells show phenotypic changes in response to altered concentrations of antibiotic even if they do not contain a tetracycline regulated gene of interest [5], [6], [10], [12], [15], we asked whether these changes may correspond to altered gene expression patterns. Indeed, using microarray analysis we found that the levels of several hundred mRNAs were altered significantly after transfer of U937T_pUHD10S cells to tetracycline free media. The mechanism through which tetR affects gene expression is unclear and may or may not involve direct interactions with DNA. Irrespectively, it is plausible that the alterations in gene expression patterns in tetR expressing U937 cells contribute to the phenotypic changes observed in these cells after tetracycline withdrawal. Moreover, even though tetR certainly does not play a physiological role in human myeloid cells like U937, several of the gene ontology terms enriched among the genes induced upon tetracycline removal correspond to biological responses that might be expected as a consequence of the experimental expression of a physiologically or pathologically relevant gene. Similarly, altered gene expression patterns may well contribute to recently reported phenotypic consequences of tetR expression in transgenic mice, which were observed even in the absence of any exogenous gene of interest regulated by it [17].

Our results also have practical implications for studies aimed at the identification of target genes of transcription factors or other genes of interest. Because of the still considerable cost of transcriptome analyses, some authors restrict their experiments to the comparison of gene of interest-containing cells maintained in the presence or absence of tetracycline. Factors of 1.5- or 2-fold are often defined as thresholds for differential gene expression, and rigorous statistical analyses including corrections for multiple hypothesis testing are frequently not applied [8], [14], [16] (and according to our experience would in fact prevent further consideration of genes whose differential expression can be confirmed by RTQ-RT-PCR). However, even after multiple hypothesis testing, 99 unique genes were deregulated more than twofold in U937T_pUHD10S cells transferred to tetracycline free media. While this number may appear small at first sight, it is large enough to significantly confound the results of target gene analyses, especially if the investigated gene of interest has few true biological targets. Furthermore, the 774 genes that are deregulated ≥1.5-fold at p<0.05 may also lead to erroneous conclusions, and may do so even in analyses in which higher stringency levels are applied: a gene that would be regulated slightly less than twofold in control cells and slightly above this threshold in gene of interest expressing cells may still not be a relevant biological target of the investigated gene. Therefore, our findings need to be taken into consideration when performing gene expression experiments using the tetR system.

## Materials and Methods

### Cell lines and cell culture

Human U937 histiocytic lymphoma cells were cultured in a humidified incubator at 37°C and 5% CO_2_ in RPMI 1640 medium (Invitrogen) containing 10% FBS (Invitrogen). To study the effects of tetracycline removal, 1 µg/ml tetracycline (Sigma) was added to the culture media for one week, and cells were then processed as described below.

U937T cells have been derived from U937 cells by stable transfection with a construct driving tetracycline-regulable expression of the tetVP16 fusion protein [5]. They were cultured in a humidified incubator at 37°C and 5% CO_2_ in RPMI 1640 medium containing 10% FBS, 0.5 µg/ml puromycin (Sigma), and 1 µg/ml tetracycline. To obtain U937T_pUHD10S cells, U937T cells were electroporated (0.17 kV, 950 µF) with the vector pUHD10S, which contains seven copies of the tetracycline operator, thus facilitating tetracycline regulable expression of its cDNA inserts [18]. Transfectants were selected, cloned, and propagated in RPMI 1640 medium supplemented with 10% FBS, 0.5 µg/ml puromycin, 1 µg/ml tetracycline, and 500 µg/ml hygromycin (PAA). To remove tetracycline from the culture media, exponentially growing cells were washed 3 times with phosphate-buffered saline (PBS) and resuspended in growth media without tetracycline. Control cultures were washed in the same manner but resuspended in media with tetracycline.

### Microarray analyses

RNA for microarray analyses was extracted from three independent replicate cultures of U937T_pUHD10S cells using the RNeasy Plus mini kit (Qiagen) according to the manufacturer's instructions. Quality control of the isolated RNA samples, labelling, and hybridization to U133 Plus 2.0 arrays (Affymetrix) were performed at the Center of Excellence for Fluorescent Bioanalytics (KFB) in Regensburg, Germany, which is an Affymetrix Service Provider and Core Facility. Sample preparation was carried out in accordance with the Affymetrix GeneChip Expression Analysis Technical Manual (one replicate) or the Affymetrix GeneChip IVT Express Kit Technical Manual (two replicates). Briefly, total RNA was reverse transcribed into double stranded cDNA, from which Biotin labeled cRNA was generated using the One-Cycle Target Labeling Kit (Affymetrix) or the 3′ IVT Express Labeling Kit (Affymetrix). The length of the cRNA products was assessed using an Agilent 2100 bioanalyzer (Agilent Technologies). Following fragmentation, cRNA products (15 µg) were hybridized to the array for 16 h at 45°C in a rotating chamber. Hybridized arrays were washed and stained in an Affymetrix Washing Station FS450 using Streptavidin-Phycoerythrin conjugate (Life Technologies) together with biotinylated anti-streptavidin antibody (Vector Laboratories), or, for the IVT protocol, preformulated Hyb, Wash, and Stain (HWS) components (Affymetrix). Fluorescent signals were measured with an Affymetrix GeneChip Scanner 3000. Primary data analysis was carried out using the Affymetrix MAS5 algorithm.

### Real time quantitative reverse transcriptase polymerase chain reaction (RTQ-RT-PCR)

Total RNA for RTQ-RT-PCR was extracted using Trizol (Invitrogen) and reverse transcribed using random hexamer primers (Invitrogen) and M-MLV reverse transcriptase (Invitrogen) according to the manufacturer's instructions. RTQ-RT-PCR was carried out in an ABI Prism 7700 Sequence Detection System (Applied Biosystems) using the Mesa Green qPCR Master Mix Plus (Eurogentec) according to the manufacturers' instructions. RTQ-RT-PCR primers are shown in Table 2. All assays were carried out in triplicate. Expression values for the genes of interest relative to the housekeeping gene *cyclophilinD* and to a reference value were determined using the ΔΔC_T_ method [19].

**Table 2:** Primers used for RTQ-RT-PCR.

Primer name	Primer sequence (5′ ->3′)
*cyclophilinD* fwd	ATATTGGAAAATGTGGAAGTGAAAGG
*cyclophilinD* rev	TCGCCAGAGCCATCTTTTG
*CD36* fwd	TGAACAGCAGCAACATTCAA
*CD36* rev	GCTGCAGGAAAGAGACTGTG
*ITGAL* fwd	CAGGAATGTATCAAGGGCAA
*ITGAL* rev	AACAGCAGCAAACTGGTACG

### Data evaluation and in silico analyses

Only probe sets with present calls in all six samples were considered for statistical analyses, which were performed on log2-transformed intensity values using the R/Bioconductor package *limma* employing a moderated t-test. Probe sets whose average expression differed at least 1.5- or 2-fold at p<0.05 between cells maintained in the presence or absence of tetracycline were considered as differentially expressed. Where indicated, multiple hypothesis testing according to the method of Benjamini and Hochberg [20] was performed, and probe sets with a false discovery rate (FDR) <10% were considered significantly deregulated. Differentially expressed unique genes were identified in the same manner, except that probe sets with no gene annotation or with gene names including the term “hypothetical” were omitted, and for probe sets sharing the same gene annotation only the most informative ones (i.e., those with the highest interquartile range) were kept. All analyses were performed using the statistical software environment R 2.10.1.

Over-represented gene ontology (GO) terms for up- or down-regulated genes were identified using DAVID [21] based on gene symbols. Up-regulated genes were functionally grouped into gene ontology networks using the Cytoscape plug-in ClueGO [22].

## Supporting Information

Table S1 Genes deregulated at least 1.5-fold at p<0.05 (Table S1A) or at an FDR <10% (Table S1B) upon withdrawal of tetracycline from U937T_pUHD10S cells. FC, fold change; FDR, false discovery rate.
(0.19 MB XLS)

Table S2 Gene ontology terms significantly overrepresented among genes deregulated ≥1.5-fold at an FDR <10% upon withdrawal of tetracycline from U937T_pUHD10S cells. Count, number of genes upregulated or downregulated in respone to tetracycline withdrawal that are assigned to the named GO term; %, percentage of genes upregulated or downregulated in respone to tetracycline withdrawal that are assigned to the named GO term. FDR, false discovery rate.
(0.04 MB XLS)
